# Troglitazone suppresses tumor cell growth and glutamine metabolism through a PPAR-independent mechanism

**DOI:** 10.1186/2049-3002-2-S1-P61

**Published:** 2014-05-28

**Authors:** Miriam Reynolds, Brian Clem

**Affiliations:** 1 Department of Biochemistry, JGBrown Cancer Center, University of Louisville, Louisville, KY 40202, USA

## Background

In tumor cells, enhanced glutamine metabolism provides anaplerotic carbon for the TCA and fatty acid synthesis as well as precursors for the production of nucleotides and glutathione. This metabolic alteration is primarily driven by disruptions in oncogenic or tumor suppressor function and ultimately results in glutamine dependency for tumor cell growth and survival. Accordingly, identification of small-molecule inhibitors targeting glutaminolysis3 may have utility as an anti-cancer therapeutic strategy. Troglitazone, a PPARγ agonist previously FDA-approved in treating Type II diabetes, has been reported to suppress glutamine metabolism. Therefore, we examined whether troglitazone treatment could disrupt glutamine metabolism and proliferation in glutamine-dependent tumor cells.

## Materials and methods

Using glutamine-dependent HeLa cervical cancer, H460 lung cancer, and RB-triple knock-out MEF cells, we characterized the effects of troglitazone treatment on: 1) cell viability, 2) glutamine metabolism by examining ^14^C-glutamine uptake, ^13^C-glutamine metabolomics, and ATP determination, and 3) expression of ASCT2, GLS, and c-Myc. To determine a PPAR requirement on these processes, siRNA and pharmacological inhibition of PPAR α or γ was performed. Rescue experiments were done through simultaneous add-back of di-methyl alpha-ketoglutarate (α-KG) or expression of a c-Myc phosphorylation mutant.

## Results

Troglitazone treatment resulted in a dose-dependent inhibition of cell proliferation and glutamine uptake that was accompanied by a decrease in ASCT2 and GLS expression. Metabolomics analysis revealed a reduction in ^13^C-glutamine incorporation into aspartate and a decrease in steady-state ATP levels, suggesting that troglitazone causes diminished glutamine anaplerosis within the TCA. Silencing PPAR α or γ activity via siRNA or a specific small molecule antagonist (GW9662) did not alter troglitazone’s effects on cell proliferation or glutamine uptake suggesting that these processes are PPAR-independent. However, troglitazone caused a dose-dependent, proteasomal-mediated decrease in c-Myc expression, while re-expression of a c-Myc phosphorylation mutant partially rescued glutamine uptake, [ATP], and cell viability. In addition, exogenous di-methyl α-KG also led to a partial rescue of ATP and cell viability. Lastly, combinatorial treatment with metformin, which sensitizes cells to glutamine disruption, resulted in a synergistic decrease in cell viability.

## Conclusions

Taken together, troglitazone may exert its anti-tumor activity in part through disruption of glutamine metabolism in a PPAR-independent manner. In addition, characterizing new anti-tumor properties of previously approved FDA therapies lends support to the possible repurposing of these agents, especially in combination with other targeted treatment strategies, such as metformin.

